# Hyperphosphorylated tau aggregation and cytotoxicity modulators screen identified prescription drugs linked to Alzheimer's disease and cognitive functions

**DOI:** 10.1038/s41598-020-73680-2

**Published:** 2020-10-06

**Authors:** Mengyu Liu, Thomas Dexheimer, Dexin Sui, Stacy Hovde, Xiexiong Deng, Roland Kwok, Daniel A. Bochar, Min-Hao Kuo

**Affiliations:** 1 Department of Biochemistry and Molecular Biology, Michigan State University, 603 Wilson Road, Room 401, Biochemistry Building, East Lansing, MI 48824 USA; 2 Department of Pharmacology and Toxicology, Michigan State University, East Lansing, MI USA; 3 Department of Biological Chemistry, University of Michigan, Ann Arbor, MI USA; 4 Department of Obstetrics and Gynecology, University of Michigan, Ann Arbor, MI USA; 5 Cayman Chemical, Ann Arbor, MI USA; 6 Present Address: Molecular, Cellular, and Developmental Biology, University of Michigan, Ann Arbor, MI 48109-1085 USA

**Keywords:** Drug discovery, Alzheimer's disease

## Abstract

The neurodegenerative Alzheimer’s disease (AD) affects more than 30 million people worldwide. There is thus far no cure or prevention for AD. Aggregation of hyperphosphorylated tau in the brain correlates with the cognitive decline of patients of AD and other neurodegenerative tauopathies. Intracerebral injection of tau aggregates isolated from tauopathy brains causes similar pathology in the recipient mice, demonstrating the pathogenic role of abnormally phosphorylated tau. Compounds controlling the aggregation of hyperphosphorylated tau therefore are probable modulators for the disease. Here we report the use of recombinant hyperphosphorylated tau (p-tau) to identify potential tauopathy therapeutics and risk factors. Hyperphosphorylation renders tau prone to aggregate and to impair cell viability. Taking advantage of these two characters of p-tau, we performed a screen of a 1280-compound library, and tested a selective group of prescription drugs in p-tau aggregation and cytotoxicity assays. R-(−)-apomorphine and raloxifene were found to be p-tau aggregation inhibitors that protected p-tau-treated cells. In contrast, a subset of benzodiazepines exacerbated p-tau cytotoxicity apparently via enhancing p-tau aggregation. R-(−)apomorphine and raloxifene have been shown to improve cognition in animals or in humans, whereas benzodiazepines were linked to increased risks of dementia. Our results demonstrate the feasibility and potential of using hyperphosphorylated tau-based assays for AD drug discovery and risk factor identification.

## Introduction

Alzheimer's disease (AD) is the most common form of adult dementia that affects 1 in 9 people 65 years and older, and 1 in 3 of those 85 years and older. Aging is the most obvious risk factor for AD. With the human lifespan extending, the number of AD patients is also on the rise, adding tremendous socioeconomical tolls to developed and developing countries. Therapeutics and preventative regimens are desperately needed to flatten the upward trend of AD cases worldwide.

The two major biomarkers for AD are senile plaques made of β-amyloid (Aβ) aggregates, and neurofibrillary tangles (NFTs) of hyperphosphorylated tau. The amyloid cascade hypothesis posits that Aβ is the initial pathological abnormality, which ultimately causes the formation of plaques and neurodegeneration^1^. Recurring failures of clinical trials of anti-Aβ measures argue strongly against a direct role of Aβ in dementia^2^. Unlike plaques, the spatiotemporal distribution of NFTs correlates with the progression of cognitive impairments^3,4^. Animal and in vitro studies support a more direct pathogenic role of hyperphosphorylated tau^5^. Indeed, neuronal and glial inclusions of abnormally phosphorylated tau also are the defining feature of about 20 neurodegenerative tauopathies that, except AD, do not display significant senile plaques^6^.

There is no prevention for AD, either. Early-onset Alzheimer's disease, which constitutes less than 10% of all AD cases, is linked to mutations in the genes involved in Aβ genesis^7^. The majority of AD cases are late-onset and sporadic. Besides aging^8^, more than 20 predisposing genetic loci have been identified in genome-wide association studies^9^. Unlike these unmodifiable predisposing loci, acquired risk factors may be more amenable targets for AD prevention. These include viral infection^10^, cerebrovascular diseases, type 2 diabetes, hypertension, obesity, dyslipidemia^11^, and such environmental factors as metals, pesticides, industrial chemicals and air pollutants^12^. Physical injuries to the brain, e.g., traumatic brain injury and chronic traumatic encephalopathy also predispose patients to AD and AD-related dementia (ADRD)^13^. Lastly, certain prescription drugs have been linked to increased risks for AD and dementia, including benzodiazepines^14^, anticholinergic drugs^15,16^ and disease-modifying anti-rheumatic drugs (DMARD)^17^. These pharmaceutical risks for AD appear to be the most easily modifiable, therefore warrant more scrutiny. The identification of multiple predisposing genetic loci and risk factors for AD suggests the involvement of different pathways in the creation of a physiological state more liable to neurodegeneration^18^. Thus far, the most likely molecular trigger for neuronal dysfunction and death appears to be the aggregation of abnormally phosphorylated tau.

Deposition of fibrillar hyperphosphorylated tau in the brain is a key biomarker of AD and tauopathies^6^. Tau is a microtubule-binding protein possibly involved in controlling axonal transport^19^, but tau homozygous knockout mice do not show significant neurodevelopmental defects^20^. In AD brain, the protein level and the phosphoryl content of tau increase by several fold^21^. Tau hyperphosphorylation is causally linked to neurodegeneration in different models^22^. However, among the 85 Ser/Thr/Tyr residues in the longest CNS tau isoform, of which at least 48 have been found to be phosphorylated in the disease tau^21,23^, it is a formidable challenge to define the phosphorylation pattern leading to the disease.

Instead of the terminal precipitates of polymeric NFTs that were once thought to overwhelm neuronal functions, accumulating evidence suggests that the oligomeric pre-tangle species of hyperphosphorylated tau plays a causal role in AD pathology. A fly model presented many transgenic tau-dependent pathological features without significant neurofibrillary tangles^24^. Intracerebral injection of tau purified from AD brain homogenates to mice caused the endogenous tau to form fibrils at areas distal to the injection site^25–27^. Animal and tissue culture studies showed that pre-tangle aggregates of hyperphosphorylated tau propagated to and damaged neighboring cells^28–32^. Hyperphosphorylated tau is preferentially secreted by exocytosis or vesicle-free mechanisms^5,33–36^. Once outside the donor cells, tau might enter the recipient cells via the action of low-density lipoprotein receptor-related protein 1 (LRP1)^37^, amyloid precursor protein (APP)^38^, or by binding directly to the membrane^39^. The exact identity of the pathogenic tau species awaits delineation. Enrichment of a 140–170 kDa oligomer is associated with memory loss in transgenic mice^40^. A pore-like structure of tau annular protofibrils was found on the membrane of human tauopathy brain samples^41^. Intermediates of tau aggregation cause leakage of artificial membrane, but longer aggregation period reduces the potency^42,43^, suggesting a window of cytotoxicity defined by the state of tau aggregation^44^.

If the pre-tangle hyperphosphorylated tau oligomer(s) is a critical pathogenic factor, drug discovery may advance more productively if a disease-relevant hyperphosphorylated tau is used as the primary subject for the initial screening. Several tau aggregation-based drug screens have been reported^45–47^. These works used unmodified tau as the primary subject to test or to screen for compounds that inhibited heparin-induced tau aggregation. One of such compounds, methylene blue, advanced to clinical trials but failed^48^. Many other tau aggregation inhibitors (TAIs) were later found to be non-selective redox modulators^49^, due apparently to the susceptibility of unmodified tau aggregation to the redox state of the two cysteine residues of tau protein^50–52^. Heparin-induced tau aggregates had a different proteinase K digestion pattern from its hyperphosphorylated counterpart from the brain^53^. It is therefore desirable to use hyperphosphorylated tau for AD drug discovery. Different means have been employed for the preparation of hyperphosphorylated tau, including in vitro kinase reactions using recombinant tau^54,55^, purification from phosphatase inhibitor-treated insect cells overexpressing human tau^56^ or isolation from human or animal model brain tissues^53,57,58^. The efficiency, yield, and the purity of the final phosphorylated tau protein were usually insufficient to support the throughput necessary for drug screen. We recently used the PIMAX approach (Protein Interaction Modules-Assisted function X)^59^ for the synthesis of hyperphosphorylated tau (p-tau) in *E. coli*^60^. This 1N4R p-tau possessed multiple phosphorylation marks for the staging of AD brain samples. Hyperphosphorylation caused tau to form cytotoxic fibrils without the heparin inducer. These unique features indicated the disease relevance of the underlying hyperphosphorylated tau, and prompted the use of this p-tau in a pilot screen of about 1300 known compounds. Results presented below show that candidates previously linked to cognitive function or to AD risks were found to control the aggregation and cytotoxicity of p-tau. A hyperphosphorylated tau aggregation and cytotoxicity-based screening platform therefore provides a sensible means to develop efficacious therapy and prevention of AD.

## Results

### Screen of a 1280-compound library identified p-tau aggregation modulators

**Figure 1:**
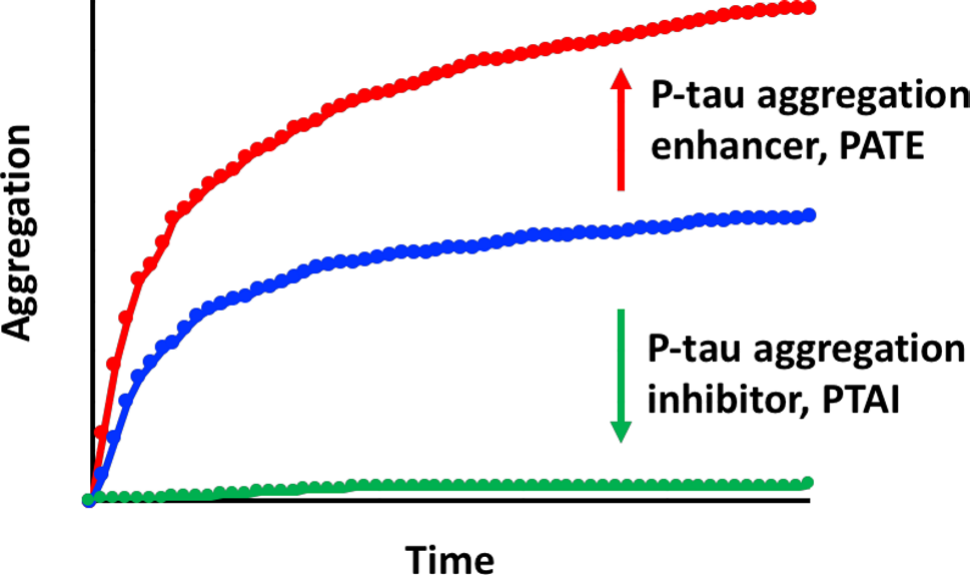
Identification of inhibitors and enhancers for the aggregation of hyperphosphorylated tau may facilitate the development of Alzheimer's disease therapy and prevention.

If the formation and propagation of hyperphosphorylated tau aggregates are the underpinning for tauopathy neurodegeneration^61^, small-molecule compounds controlling these molecular events may deter or expedite the progression of cognitive impairments. The former would be candidates for therapeutics development, while the latter may be manageable risk factors (Fig. 1). As described in “Introduction” above, we have produced hyperphosphorylated 1N4R tau (p-tau) in *E. coli* that possessed AD pathology-associated phosphoepitopes, and formed cytotoxic aggregates without an inducer^60^. These characters were absent in unmodified tau, suggesting the possibility of identifying new categories of compounds for the control of hyperphosphorylation-driven behaviors of tau.

**Figure 2:**
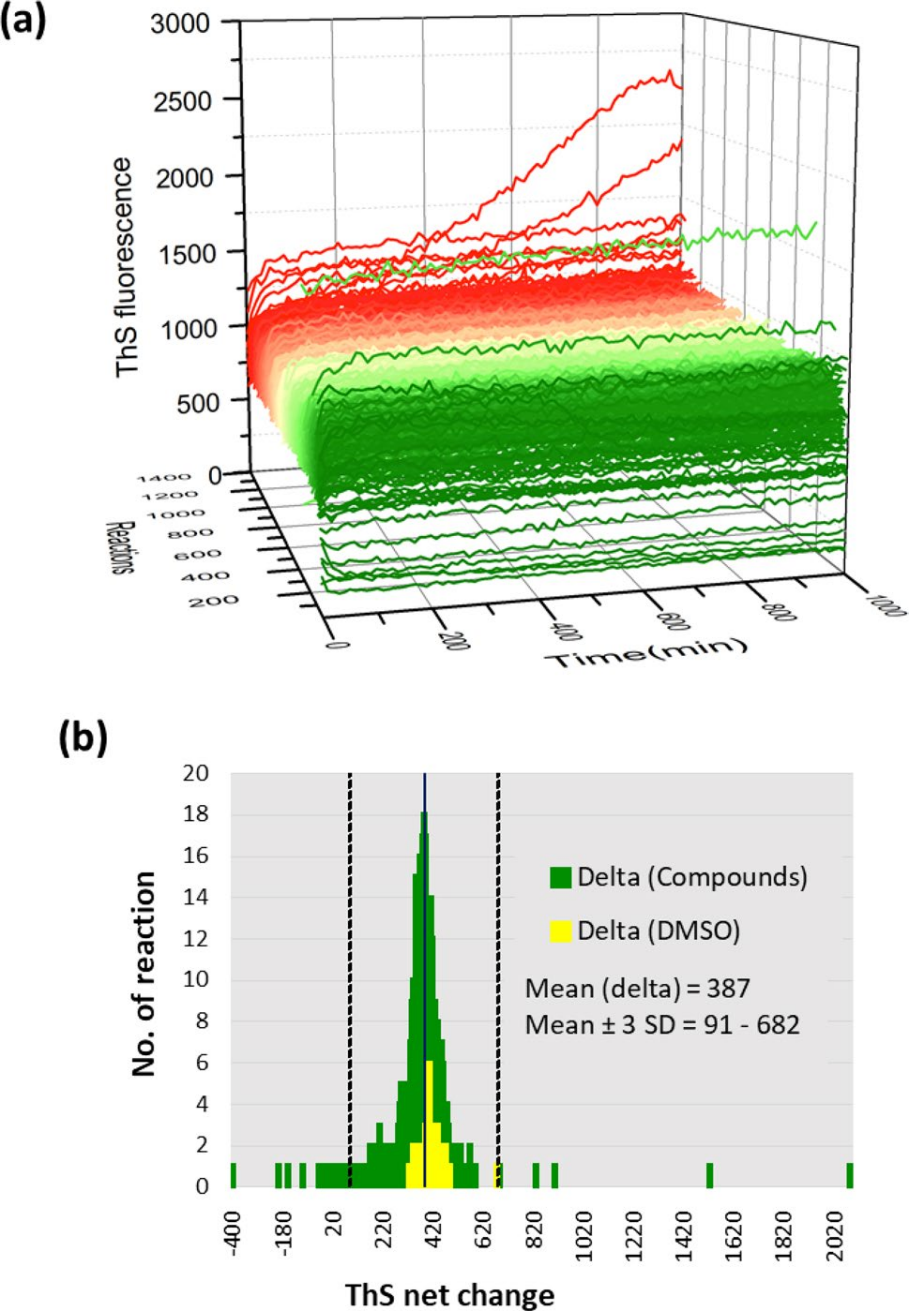
Pilot screen of a 1280-compound library for p-tau aggregation modulators. (**a**) Aggregation curves of 6-µM 1N4R p-tau in the presence of 30-µM compounds from the library or DMSO (dimethyl sulfoxide) vehicle control. Kinetic curves were arranged with ascending ThS net change (delta) values from green to red. (**b**) Frequency distribution of ThS fluorescence net changes over the course of 16-h assays. "Delta" in the graph refers to the average net difference of ThS fluorescence of reactions with the library compounds (green) or only the DMSO vehicle control (yellow). The three vertical lines represent the mean and ± 3 SD (standard deviation) of all reactions with the compounds.

To test the feasibility of applying hyperphosphorylated tau to AD drug discovery, we conducted a proof-of-principle pilot screen of a commercial 1280-compound library (95% were approved drugs). Aggregation of 6-µM p-tau under the influence of 30 µM of each of the library compounds or a DMSO vehicle control was monitored in 384-well plates every 10 min for ThS fluorescence changes over 16 h. The full-course aggregation curves of all reactions are shown in Fig. 2a. To assess the quality of the high-throughput p-tau aggregation reactions, we used the T_0_ and T_16hr_ ThS fluorescence units of no-compound control reactions to calculate the Z′-factor and coefficient of variation (CV), two commonly used criteria for robustness of high-throughput screens (HTS)^62^. A Z′-factor greater than 0.5 and CV smaller than 10% indicate robustness^63^. In the case of p-tau aggregation reactions, the Z′-factor and CV were 0.699, and 8.9%, respectively, both exceeding the expectation. To identify compounds with significant p-tau aggregation modulation, the net change of ThS fluorescence was calculated from all reactions (see Supplemental Table 1 for complete listing). The frequency plot (Fig. 2b) summarizes the distribution of these reactions. All compounds deviating 3 standard deviations (SD) or more from the mean, and a small number of selective ones near 2 SD from the mean were subjected to dose–response curve (DRC) tests (1.625–100 µM). Those showing p-tau aggregation modulation activity in DRC were acquired from commercial sources for verification. Nine p-tau aggregation inhibitors (PTAI) and two enhancers (PTAE) were confirmed (see Supplemental Fig. 1 for structures). Table 1 shows these 11 compounds. Quantification of the concentration of a compound needed to reduce aggregation of 6-µM p-tau by 50% (i.e., EC_50_) revealed a range from 8 to 68 µM among the 9 PTAIs.

**Table 1:** Summary of p-tau aggregation inhibitors and enhancers identified in the library screen. EC_50_ was defined as the concentration of each PTAI that reduced the aggregation of 6-µM p-tau to 50% of the maximal. NA, not applicable. *Hexachlorophene consistently showed PTAI activity but was inconsistent in protecting cells from p-tau toxicity.

	EC50 (µM)	p-tau cytotox	Medical uses
Apomorphine	18.1	Reduced	Parkinson's disease
Clofazimine	68	No change	Leprosy
Hexachlorophene	17.8	Inconclusive*	Disinfectant
Hydralazine	21.8	No change	Hypertension
Idebenone	10.5	Reduced	Varying, but currently inactive
Itraconazole	10.6	No change	Anti-fungal
Nifedipine	24.3	No change	Hypertension
Nisoldipine	8	No change	Hypertension
Raloxifene	31.2	Reduced	Osteoporosis
Carmofur	NA	Enhanced	Antineoplastic
Prednicarbate	NA	Enhanced	Skin conditions

### R-(−)apomorphine and raloxifene are p-tau aggregation inhibitors and protect cells

Of the 9 PTAIs, R-(−)apomorphine and raloxifene were selected for further studies for three reasons. Firstly, we took advantage of the cytotoxicity of p-tau^60^ (detailed below) to evaluate whether these compounds modulated p-tau cytotoxicity without the use of the fibril dye, ThS, or any inducer. Three PTAIs [R-(−)apomorphine, idebenone and raloxifene] reduced p-tau cytotoxicity and both PTAEs showed enhancement (Table 1 and Supplemental Fig. 2). PTAIs without an apparent effect on p-tau cytotoxicity were set aside. Secondly, R-(−)apomorphine and raloxifene can pass the blood brain barrier. R-(−)apomorphine is a dopamine receptor agonist used to alleviate symptoms of Parkinson's disease^64^. Raloxifene is a selective estrogen receptor modulator (SERM) that has been shown to penetrate the blood brain barrier^65^. The likelihood of developing effective therapeutics from these two compounds is therefore higher. In contrast, idebenone, being a potent antioxidant and high lipophilicity, has very low bioavailability^66^. Lastly, both R-(−)apomorphine and raloxifene have been linked to the preservation of cognition^67,68^ (detailed in “Discussion” below), suggesting that these prior observations might be related to the control of NFT in the brain.

**Figure 3:**
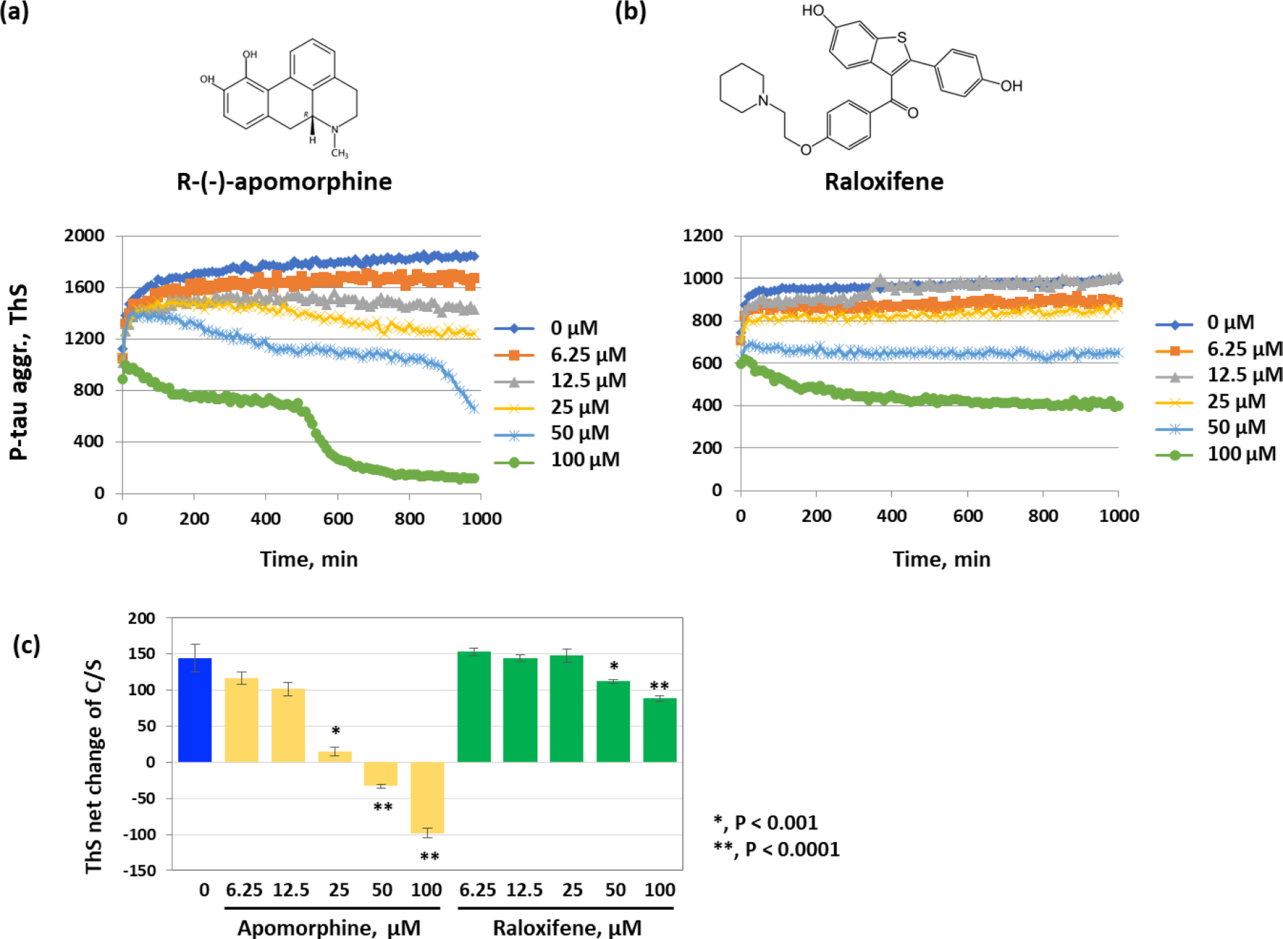
R-(−)-apomorphine and raloxifene are p-tau aggregation inhibitors (PTAIs) that act independently of the redox state of p-tau. (**a**) and (**b**) P-tau aggregation inhibition dose response curves of R-(−)-apomorphine and raloxifene. Aggregation of 6 µM of p-tau was done in the presence of the indicated concentrations of either compound. (**c**) Both R-(−)-apomorphine and raloxifene act independently of the two cysteine residues of p-tau. P-tau aggregation reactions with the C291S C322S (C/S) mutant p-tau were done in the presence of the indicated amounts of either compound. Shown are net changes of ThS over 16 h. R-(−)-apomorphine repetitively shows negative ThS net change at high doses.

To gain a better understanding of the potential of R-(−)apomorphine and raloxifene for AD drug development, we first compared the kinetics of p-tau aggregation inhibition by these two chemicals. Figure 3 shows different patterns. The action of R-(−)apomorphine consisted of multiple phases: a lag phase in which ThS fluorescence increased at the "normal" rate briefly, followed by stabilization or a slow reduction, then a sharp decline. Eventually, the highest dose of R-(−)-apomorphine caused the ThS fluorescence to drop well below the initial signal. At the same dosages, on the other hand, raloxifene elicited a typical inhibitory pattern by preventing the elevation of ThS fluorescence. P-tau aggregation appeared to be stalled from the beginning by 50 and 100 µM of the inhibitor. The distinctions in kinetics suggested that R-(−)-apomorphine and raloxifene targeted different aspects of p-tau fibrillogenesis.

Many “hits” from prior tau-based HTS were non-selective redox modulators^49^, due most likely to the susceptibility of unmodified tau fibrils to the oxidation of two cysteine residues of tau, C291 and C322. Because p-tau aggregation was not affected by changing the two cysteine residues to alanine or serine^60^, we suspected that R-(−)-apomorphine and raloxifene would act independently of these two cysteine. Figure 3c and Supplemental Fig. 3a show that the aggregation of the C/A or C/S mutant p-tau was effectively inhibited by either compound. By itself, the C/S p-tau gained 130 units of ThS fluorescence over the course of the aggregation reaction (blue column). With increasing doses of either compound, the net gain of ThS fluorescence decreased progressively. The extent of reduction mimicked that from the use of the wildtype p-tau (Fig. 3a,b) in that higher doses of R-(−)-apomorphine caused the ThS signals to drop below the initial level. Raloxifene, on the other hand, showed milder but still easily recognizable inhibition. It was therefore concluded that R-(−)-apomorphine and raloxifene antagonized p-tau aggregation in a mechanism(s) not related to the two cysteine residues of p-tau.

**Figure 4:**
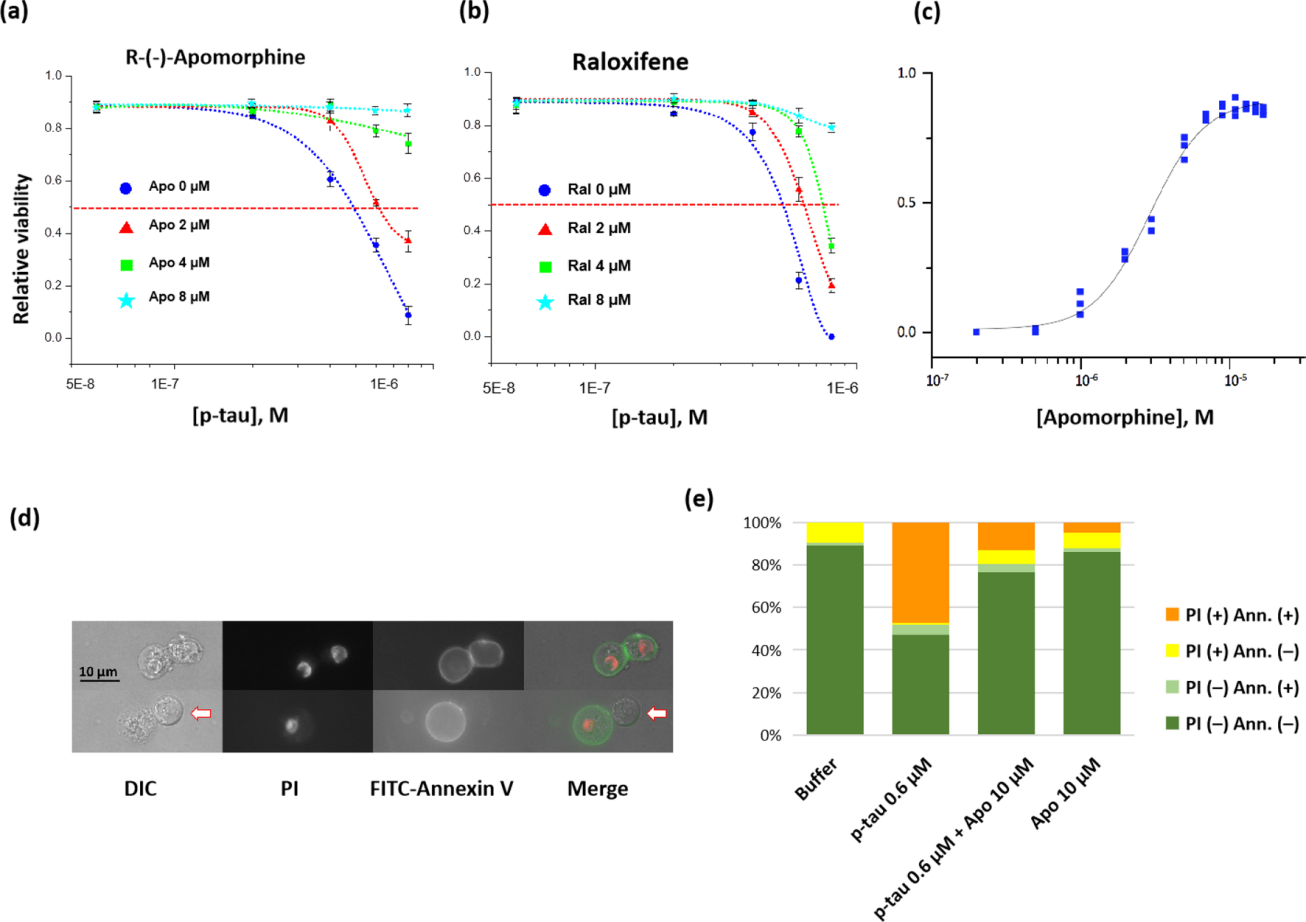
Both R-(−)-apomorphine and raloxifene protect cells from p-tau cytotoxicity. (**a**) and (**b**) Viability curves of SH-SY5Y cells treated with varying concentrations of p-tau and (**a**) R-(−)-apomorphine or (**b**) raloxifene. P-tau (without pre-aggregation) and compounds were added together to cells. Cell viability was quantified by FDA and PI differential staining 24 h later. The red dash lines indicate 50% viability, with which LD_50_ was derived. (**c**) Estimation of EC_50_ of R-(−)-apomorphine. SH-SY5Y cells were treated with 0.8-µM p-tau in the presence of 0–17 µM of R-(−)-apomorphine. Cellular viability was characterized as above and plotted against the compound concentration. Trend lines in panels (**a**)–(**c**) were obtained with the dose response curve fitting function of the software Origin (Origin Pro 8, OriginLab Corporation, Northampton, MA, USA). (**d**) Representative images of apoptotic cells after treatment of p-tau. SH-SY5Y cells were treated with 0.6 µM of p-tau for 12 h before harvesting for annexin V and PI staining. Under this sub-lethal dose of p-tau, apoptotic and live cells were easily detectable, allowing for the assessment of the effect of R-(−)-apomorphine. The white arrow shows a live cell with normal morphology. (**e**) R-(−)-apomorphine curtails the pro-apoptotic activity of p-tau. The percentage of apoptotic SH-SY5Y cells was quantified by annexin V and PI double staining of cells receiving 12 h of the indicated treatment under each column in the graph. Error bars are standard deviations; n = 3. Apo, R-(−)-apomorphine; Ann, annexin-V; PI, propidium iodide.

Another unique character of p-tau that is relevant to AD pathology is that p-tau causes cell death at sub-micromolar concentrations^60^ (see below). If this cytotoxicity is part of the underlying reasons for neurodegeneration, PTAIs that quenched p-tau cytotoxicity would be better candidates to be funneled through further development. To examine whether R-(−)-apomorphine or raloxifene could protect cells from p-tau, the neuroblastoma SH-SY5Y cells were treated with different doses of p-tau for 16–20 h before propidium iodide (PI) and fluorescein diacetate (FDA) staining and microscopic inspection. FDA is converted by cellular esterases into a fluorogenic fluorescein, therefore revealing live cells. PI is excluded by living cells, but diffuses into dead cells where it binds nucleic acid and emits fluorescence. FDA/PI differential staining thus provides a quantitative assessment of cellular viability. The blue curves in Fig. 4a,b depict the cellular viability after treatment with 0–0.8 µM of p-tau. The LD_50_ of p-tau (concentration of p-tau causing 50% cell death) was found to be around 0.5 µM for this cell line (red horizontal lines). Co-incubating p-tau with increasing amounts of R-(−)-apomorphine or raloxifene caused the LD_50_ values of p-tau to increase progressively, demonstrating p-tau cytotoxicity attenuation. The more potent cytoprotection by R-(−)-apomorphine was consistent with its stronger power in inhibiting p-tau aggregation (Fig. 3). To obtain additional quantitative assessment of the observed cytoprotection, SH-SY5Y cells were again treated with a fixed dose of p-tau (0.8 µM) along with different amounts of R-(−)-apomorphine. The resultant cellular viability was plotted as a function of R-(−)-apomorphine dosages to determine EC_50_, the concentration of a compound to reduce p-tau cytotoxicity by 50%. In the two independent assays, the EC_50_ of R-(−)-apomorphine against 0.8 µM of p-tau was found to be 2.9 and 3.4 µM (Fig. 4c and data not shown). Under the same condition, the EC_50_ of raloxifene was 4.8 µM (data not shown), again manifesting that both PTAIs antagonized the cytotoxicity of p-tau, with the former being more potent. In addition, the C/A and C/S mutants that could not go through Cys-Cys disulfide bond formation were also tested for their effects on cell survival in the absence or presence of R-(−)-apomorphine and raloxifene. Supplemental Figs. 3b,c demonstrated the reduction of p-tau C/A and C/S cytotoxicity by these two compounds. Again, R-(−)-apomorphine exhibited more potent protective power than did raloxifene.

One likely mechanism for p-tau-inflicted cell death was apoptosis^60^, evidenced by cellular staining of an apoptosis marker, annexin V (Fig. 4d). Cells treated with p-tau showed a significant increase staining of annexin V and PI. Microscopic quantification showed that without p-tau, 90% of cells were viable and were refractory to annexin V staining (first column, Fig. 4e). Treatment with 0.6 µM of p-tau caused 47% of cells stained positive with both annexin V and PI (orange sector, second column). A small number of cells were annexin V-positive but PI-negative, suggesting early apoptosis. In the presence of 10 µM of R-(−)-apomorphine, the live and non-apoptotic cells increased from 47 to 77% (P < 0.001). The reduced apoptotic population was consistent with the overall improved cell viability shown in Fig. 4a,c.

**Figure 5:**
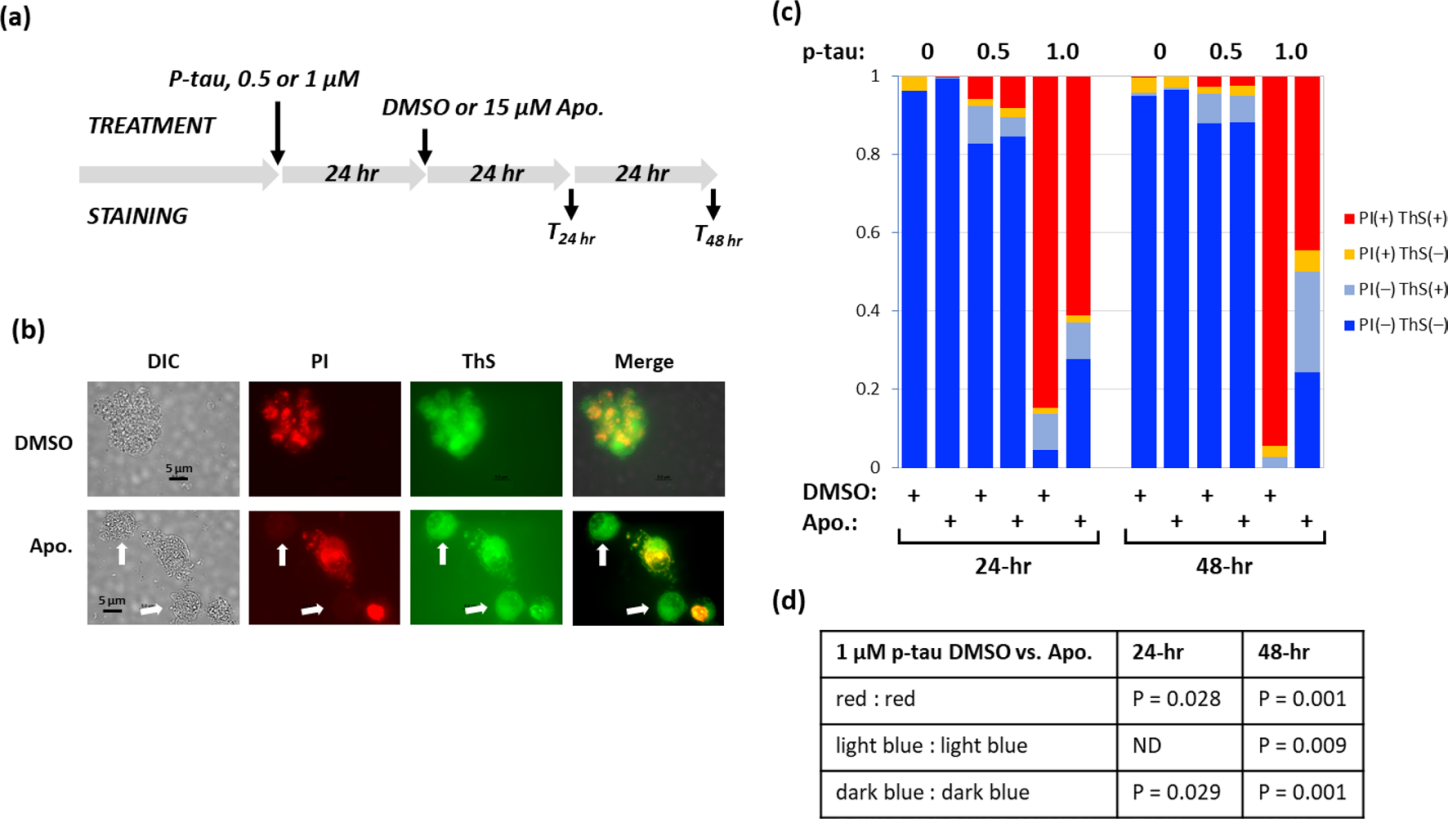
R-(−)-apomorphine reduces p-tau-induced intracellular fibrils buildup and cell death. (**a**) Experimental design. (**b**) P-tau treated HEK293T cells supplemented with DMSO or R-(−)-apomorphine were stained by ThS (thioflavin S) and PI and visualized microscopically. The majority of cells in the DMSO control group were stained positive by both dyes, indicating an increase in cell death with intracellular fibril buildup. R-(−)-apomorphine treatment increased the number of viable cells with ThS fluorescence. Two ThS ( +) PI (−) cells are marked with white arrows. These images were from T_24hr_ time point in panel (**a**) with 0.5 µM p-tau treatment. (**c**) Quantification of ThS and PI stainability among cells. Cells were categorized based on one of the four ThS/PI double-staining patterns. The total number of cells captured microscopically under each treatment was used as the denominator for the calculation of the proportion of each staining pattern, and expressed in this stacked column chart. (**d**) The standard deviations from data of panel (**c**) (n = 3) were used to calculate the P values by one-tailed Student's t tests to compare DMSO and R-(−)-apomorphine treatment under each condition. ND, not different. n = 3.

We next examined whether R-(−)-apomorphine could benefit cells after p-tau assault had begun. Figure 5a shows the assay workflow. Fifteen micromolar of R-(−)-apomorphine or DMSO was added to cells that had been exposed to 0.5 or 1 µm of p-tau for 24 h. Cells were harvested for ThS/PI staining after additional 24 or 48 h. ThS staining helped visualize intracellular fibrils^45^. In this experiment, we used HEK293T cells to avoid potential interference from the endogenous tau. A quick microscopic survey revealed that p-tau treatment caused many cells to be stained by both PI and ThS (DMSO panel, Fig. 5b), indicating cell death with cytoplasmic fibrils buildup. Adding R-(−)-apomorphine 24 h after the initial p-tau attack resulted in a visible increase of viable cells (i.e., free of PI staining); some of these cells remained to be stainable by ThS (white arrows, Fig. 5b). To gain a quantitative assessment, the numbers of the four possible combinations of ThS/PI staining were tallied. Figure 5c shows that most p-tau-treated cells were dead with cytoplasmic ThS staining (red columns; 85% in 24-h, 94% in 48-h DMSO samples). However, R-(−)-apomorphine effectively reduced the number of PI ( +) ThS ( +) cells (24 h: 85% to 61%, *P* = 0.028; 48 h: 94% to 44%, *P* = 0.001) even though this compound was given 24 h after p-tau had started its action. Of the 48-h treatment samples, half of the PI (-), viable population were free of the ThS signal (dark blue bars; *P* = 0.001 when compared with the DMSO group), suggesting enhanced clearance and/or blocked absorption of p-tau. Intriguingly, live cells with cytoplasmic ThS fluorescence also increased from 3 to 26% in the 48-h samples (light blue bars; *p* = 0.009), suggesting better cellular tolerance to p-tau aggregates, or that the remaining fibrils revealed by ThS fluorescence were less toxic in the presence of R-(−)-apomorphine.

Together, cell-based assays shown in Figs. 4 and 5 revealed strong correlation between cytoprotection and anti-aggregation by R-(−)-apomorphine and raloxifene. These results may provide a molecular explanation for the reported cognitive protection associated with these two compounds^67,68^.

### Selective benzodiazepine drugs enhance p-tau aggregation and cytotoxicity

**Figure 6:**
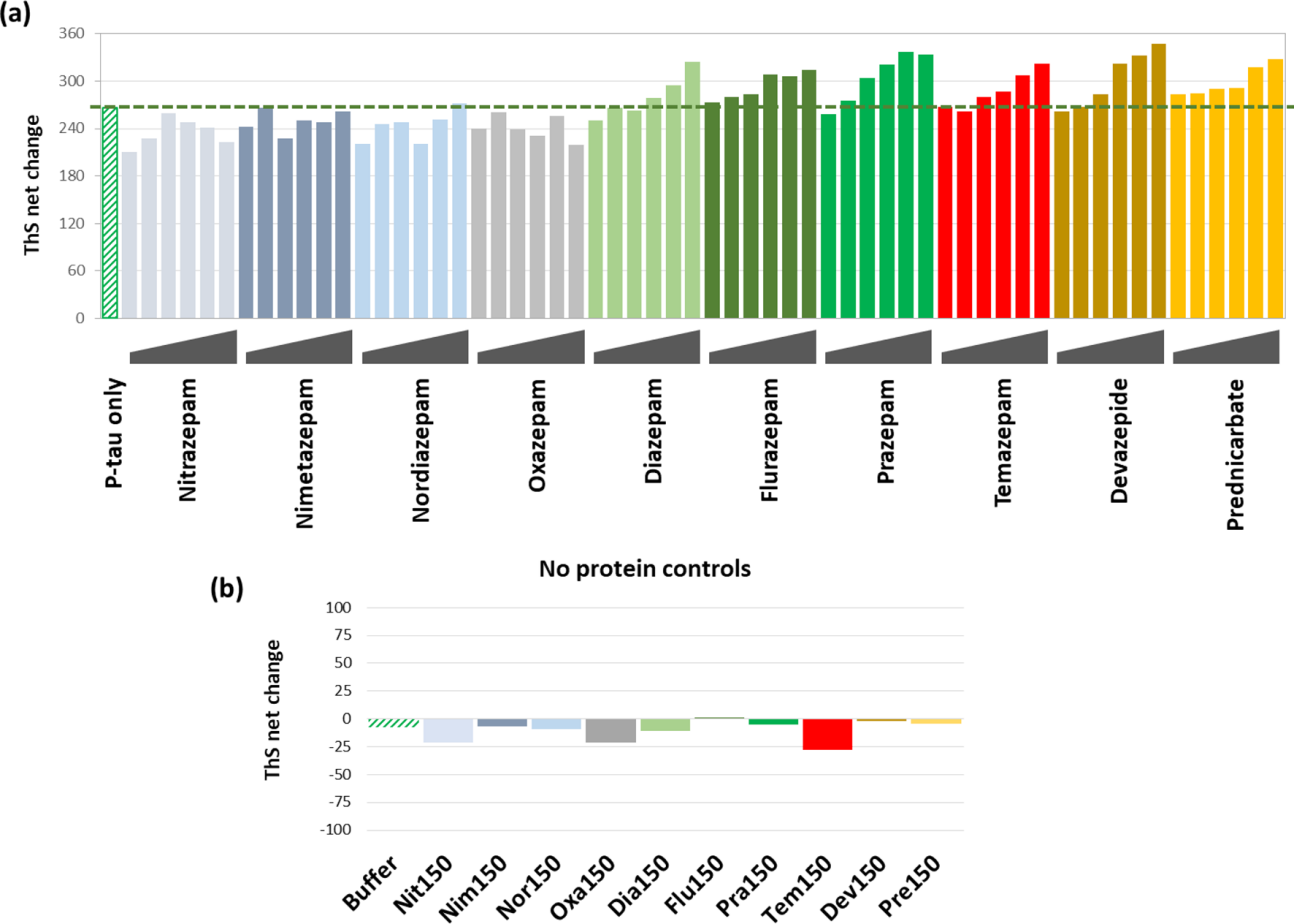
Selective prescription benzodiazepines are p-tau aggregation enhancers (PTAEs). (**a**) Net changes of ThS fluorescence from p-tau aggregation under the influence of 6.25, 12.5, 25, 50, 100 and 150 µM of benzodiazepines. Prednicarbate, a synthetic steroid, served as a PTAE control (yellow bars). (**b**) Benzodiazepine and prednicarbate did not elevate ThS fluorescence without p-tau. Shown are net changes of ThS fluorescence after 16 h of incubation with 150 µM of each of the indicated compounds, indicated by the first three letters of each compound.

Given the tremendous socioeconomical burdens of AD, measures that prevent or delay the onset of the devastating neurodegeneration of AD will be highly impactful. If hyperphosphorylated tau-inflicted neuronal damages are an underpinning for AD and other tauopathies, enhancers of p-tau aggregation (PTAEs) may be risk factors for neurodegeneration. The library screen mentioned above identified prednicarbate and carmofur as PTAEs (see Supplemental Fig. 1 for structures). Prednicarbate is a synthetic corticosteroid for dermatological use, and carmofur (1-hexylcarbamoyl-5-fluorouracil) is an anti-neoplastic pyrimidine analogue. While both compounds appeared to exacerbate p-tau cytotoxicity (Supplemental Fig. 2b), these drugs are unlikely to pose a significant neurodegeneration threat to the general population due to their primary medical use. On the other hand, epidemiological studies reported increased risks of dementia from long-term use of benzodiazepine drugs (BZDs)^14,69^. BZDs are among the most widely prescribed medication, in particular to the elderly, for depression and insomnia^70,71^. We hypothesized that certain BZDs acted as a PTAE to potentiate p-tau aggregation and cytotoxicity. The compound library used in the aforementioned screen did not include BZD family drugs. We therefore randomly picked nine prescription BZDs for p-tau aggregation assessment (Fig. 6; see Supplemental Fig. 5 for chemical structures). To 6-µM p-tau aggregation reactions, increasing doses of each of these BZDs were added. Comparison of the 16-h net changes of ThS fluorescence showed that prazepam, flurazepam, temazepam, diazepam, and devazepide enhanced p-tau aggregation in a dose-dependent manner, whereas nitrazepam, nimetazepam, oxazepam, and nordiazepam did not (Fig. 6a). None of these compounds raised ThS fluorescence by themselves (Fig. 6b). It was therefore concluded that selective BZDs were able to stimulate p-tau aggregation in vitro.

**Figure 7:**
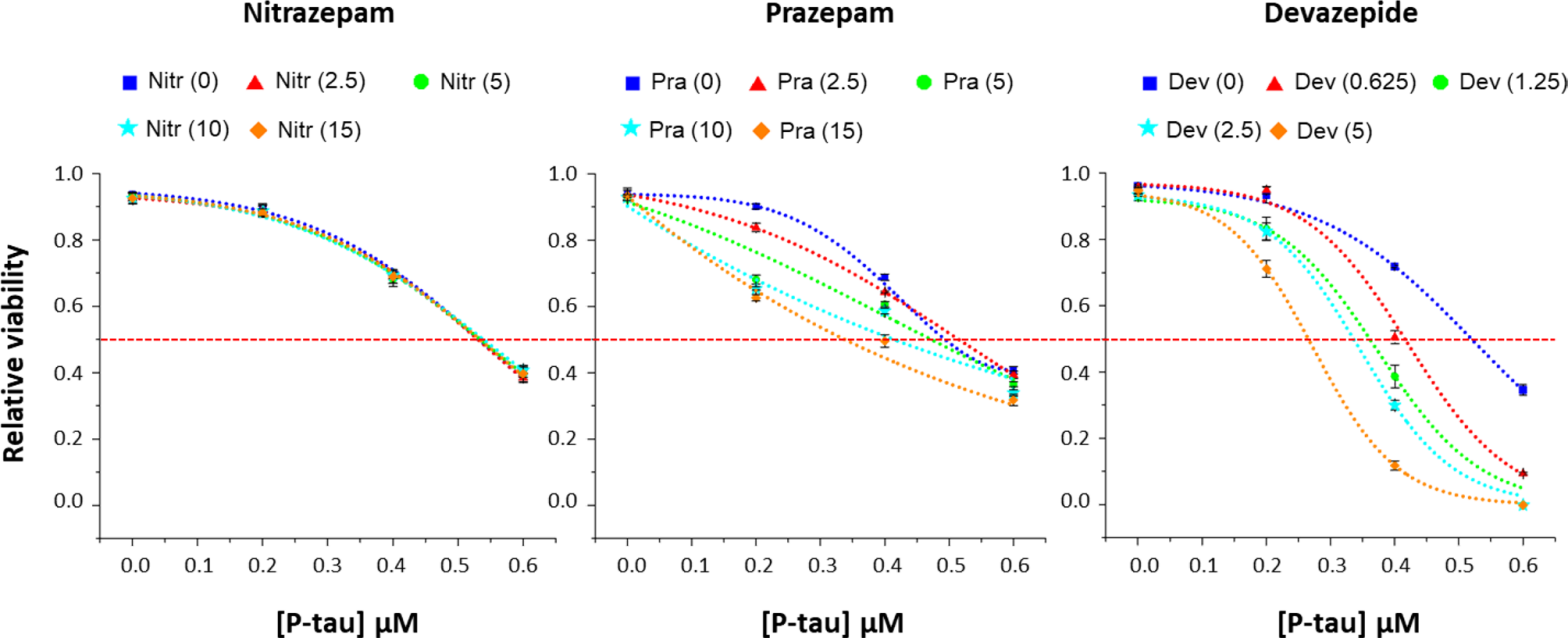
Enhanced cytotoxicity of p-tau by benzodiazepines with the PTAE activity. SH-SY5Y cells were treated with 0–0.6 µM of p-tau in conjunction with varying doses of nitrazepam, prazepam or devazepide. Devazepide consistently showed stronger exacerbation on p-tau cytotoxicity, hence lower concentrations were used here. The red dotted lines indicate 50% viability. Curve fitting was done with the dose–response fitting function of the software Origin (version 8, OriginLab Inc.). The number in parentheses represents the concentration (in µM) of the indicated compound.

To see whether the PTAE activity of BZDs would lead to enhancement of p-tau-inflicted cell death, SH-SY5Y cells were treated with p-tau pre-incubated with different concentrations of nitrazepam (a non-PTAE), prazepam or devazepide. P-tau caused cell death (blue squares, Fig. 7) with an LD_50_ value of approximately 0.54 µM. Without p-tau, none of the three BZDs affected cellular viability (i.e., 0-µM p-tau). The curve of cell death shifted progressively to the left (i.e., decreasing LD_50_) when increasing concentrations of prazepam and devazepide were present. Between these two drugs, devazepide consistently showed stronger PTAE activity (data not shown), which was in excellent agreement with its stronger p-tau cytotoxicity enhancement (note the use of lower concentrations of devazepide). In contrast, nitrazepam, which had no PTAE activity, did not alter the cytotoxicity of p-tau at all. The five cell death curves with 0 to 15 µM of nitrazepam were indistinguishable from each other. Together, biochemical and cell-based experiments in Figs. 6, 7 and Supplemental Fig. 2b demonstrated that compounds that enhanced the in vitro aggregation of p-tau might also exacerbate the cytotoxicity associated with this protein, suggesting a possible explanation for increased AD risks by BZD drugs.

## Discussion

According to the World Health Organization, 50 million people in the world are living with dementia, of which 2/3 are Alzheimer's disease. Finding an effective therapeutic or prophylactic regimen for AD will have tremendous health and socioeconomical impacts. This work presents proof-of-principle evidence for a novel, hyperphosphorylated tau-based platform supporting AD drug discovery and risk factor initial assessments. The center of this platform is p-tau produced by the PIMAX approach. We have recently reported that p-tau produced by PIMAX possesses phosphorylation marks intimately associated with cognitive impairments in AD patients^60^. Hyperphosphorylation apparently renders tau prone to form cytotoxic aggregates that trigger apoptosis and cell death in a manner that does not require an inducer, such as heparin. The character of heparin-independent aggregation and cytotoxicity is critically advantageous over the unmodified tau that has been used in multiple futile drug screens^45–49^, for the pathophysiological relevance of heparin in AD remains elusive. Compounds identified in a tau-based aggregation primary screen may contain a large number of molecules that act on the interface between tau and heparin, not tau aggregates per se. In addition, changing the two cysteine residues to either alanine or to serine does not impact the inducer-independent aggregation of PIMAX p-tau^60^, suggesting that non-specific redox modulators are unlikely to cause false positive results in high-throughput screens, as they did for unmodified tau-based drug screens^49^. Using a combination of inducer-free aggregation and cell assays, we identified modulators for both p-tau aggregation and cytotoxicity. Both p-tau aggregation inhibitors and enhancers were identified, and that, importantly, these compounds have previously linked to dementia and Alzheimer's disease. The assay platform described in this work therefore holds a promising prospect for exploration of AD disease-modifying compounds.

The current AD drug discovery field has been dominated by Aβ-centric strategies and trials with limited successes^2^. The trial failures and the fact that NFTs, but not Aβ, predict cognitive decline, led to heightened interests in NFT-targeting drugs. Unfortunately, such endeavors also encountered setbacks^49,72^. One possibility is that the subject proteins used for tau aggregation inhibitor (TAI) screens lack the hyperphosphorylation mark that is likely key to converting a normal tau to one that not only forms fibrils but also propagates pathology between cells^73^. The inducer-free aggregation and substantial impacts on cell viability of hyperphosphorylated tau produced by PIMAX suggest that TAIs, identified from heparin-induced tau aggregation assays, may not have the same inhibitory effect on p-tau aggregation. This appears to be the case. For example, two TAIs found in a high-throughput screen using heparin-induced K18 P301L aggregation as the primary assay^49^ were subjected to heparin-free p-tau aggregation reactions (Supplemental Fig. 4). At 50 or 200 µM, compound 1 had no effect on p-tau aggregation (Supplemental Fig. 4a), whereas compound 2 showed moderate inhibition. However, even when the doses were raised to 400 µM or 1 mM, p-tau aggregation was reduced to only 57% and 46%, respectively. In comparison, 50 µM of raloxifene completely blocked p-tau aggregation (Fig. 4b). These results demonstrated the unique nature of tau aggregation resulting from hyperphosphorylation, and that using p-tau aggregation as the primary assay for drug screen may uncover compounds that might escape unmodified tau-based screens.

For high-throughput drug screening, p-tau cytotoxicity appears to be an effective orthogonal assay following the aggregation modulator screen. Firstly, cell-based assays were done without heparin or ThS. Molecules that affect heparin or ThS action but not p-tau aggregation per se will likely be ruled out by the lack of influence on p-tau cytotoxicity. Secondly, using the ability of p-tau to damage tissue culture cells as a model for neurodegeneration, compounds that alter p-tau cytotoxicity are therefore better connected to AD onset and progression. From 1280 compounds in the chemical library, 9 PTAIs and 2 PTAEs were found in the initial screen and the subsequent secondary DRC tests (Table 1). When they were tested in cell viability assays, only R-(−)-apomorphine, raloxifene, and idebenone showed consistent cytoprotection (Supplemental Fig. 2a). Hexachlorophene showed inconsistent results in cell protection assays, due likely to its high propensity to oxidation (data not shown). Idebenone is a coenzyme Q analogue with limited bioavailability^66^. Prednicarbate, a PTAE, appeared to enhance the cell killing by p-tau, while carmofur exhibited borderline effects (Supplemental Fig. 2b). Prednicarbate is a synthetic corticosteroid for dermatological use^74^. Carmofur (1-hexylcarbamoyl-5-fluorouracil) is an anti-neoplastic pyrimidine analogue that inhibits acid ceramidase upregulated in many tumors^75^. These four compounds, while having limited implications in AD, may provide a useful tool to study the mechanisms by which p-tau aggregation and cytotoxicity might be controlled.

R-(−)-apomorphine and raloxifene hold a promise for further AD therapeutics development due to their brain permeability and association with cognitive preservation. R-(−)-apomorphine is a dopamine receptor agonist, whereas raloxifene is a selective estrogen receptor modulator (SERM). Dopamine and several other SERMs including tamoxifen and diethylstilbestrol were present in the pilot screen library analyzed above, but none of them affected p-tau aggregation (see Supplemental Table 1), suggesting additional structural features unique to these two compounds for the observed PTAI activity. In a 3xTg mice (APP Swedish, MAPT P301L, PSEN1 M146V) study^67^, R-(−)-apomorphine elevated levels of the insulin-degrading enzyme and reduced Aβ, resulting in memory improvement. Given the link between R-(−)-apomorphine and p-tau, it will be very interesting to see whether this drug also antagonizes tau aggregation in 3xTg mice. Clinically, R-(−)-apomorphine has a potent emetic property and is administered subcutaneously as acute, intermittent treatment of the "off" episodes associated with advanced Parkinson's disease. This drug is therefore not ideal for AD treatment. On the other hand, it has been reported that a spike of tau hyperphosphorylation happens after brain injury^76^ and general anesthesia^77^, due likely to acutely diminished phosphatase activities^78,79^. Whether this transient tau hyperphosphorylation is related to the positive association between dementia and traumatic brain injury^13^ or exposure to general anesthesia^80^ remains an open question. Animal studies may provide a clue as to whether R-(−)-apomorphine can be used to neutralize hyperphosphorylated tau following brain injury or general anesthesia exposure, thus reducing the likelihood of AD and ADRD after recovery.

Raloxifene is used to treat osteoporosis of postmenopausal women^81^. A randomized, placebo-controlled, 3-year trial on 5386 participants (mean age 66.3 years, range 35.7–80.9) showed reduced risks of mild cognitive impairment (MCI) among women who received a daily dose of 120 mg of raloxifene^68^. However, another 12-month trial involving 42 women (mean age 76 years, range 68–84) with late-onset AD of mild to moderate severity showed no significant benefits of raloxifene for AD treatment^82^. Some of the key differences between these two studies, such as the age (66.3 vs. 76 years) and the initial cognitive status (mostly cognitively normal vs. mild-to-moderate severity of AD) of the participants, may underlie the deviating outcomes, and suggest that raloxifene may only be effective when given at an early stage of cognitive decline.

It remains a formidable challenge to identify early AD patients for trials or treatment. Preventative measures against modifiable risk factors appears to be a more manageable approach at the present time. Several benzodiazepines were found to be enhancers for p-tau aggregation. The biochemical PTAE activity of BZDs, or the lack thereof, correlated with changes in p-tau cytotoxicity (Figs. 6, 7). Interestingly, the cytotoxicity enhancement activity of these BZDs required pre-incubation with p-tau (24 or 48 h; data not shown), suggesting that the observed exacerbation of cell death was through the production of a more potent p-tau toxin, not via the known targets of BZDs, which include the GABA_A_ receptor^83^ and the mitochondrial outer membrane translocator protein TSPO^84^.

Besides BZDs, certain anticholinergic drugs^15,16^ and DMARD (disease modifying anti-rheumatic drugs)^17^ were also linked to increased risks of dementia. Some of these drugs were included in the library used for the pilot screen above, but had insignificant effects on the aggregation of the 1N4R p-tau used in this study (Supplemental Table 1). While these drugs may promote dementia through a molecular target unrelated to tau, it remains formally possible that other isoforms of tau (e.g., 4R vs. 3R) phosphorylated by another kinase (e.g., CDK5) may be more responsive to some of these drugs. A panel of different p-tau species will therefore provide a valuable tool for more comprehensive studies.

In conclusion, this work presents proof-of-principle evidence for a novel, hyperphosphorylated tau-based AD drug and risk factor screening platform. This platform can be scaled up for high-throughput screens of a much larger number of compounds, and can also be applied to more targeted categories of molecules to enable drug repurposing and risk factor assessment, thereby facilitating the development of therapeutic and preventive regimens for Alzheimer's disease.

## Materials and methods

### Materials

Prestwick FDA Approved Chemical Library was provided by MSU Assay Development and Drug Repurposing Core (ADDRC). R-(−)-apomorphine hydrochloride, raloxifene hydrochloride, fosinopril, nifedipine, nisoldipine, idebenone, itraconazole, prednicarbate, carmofur, diazepam, flurazepam, temazepam, prazepam, nitrazepam, nimetazepam, nordiazepam and oxazepam were from Cayman Chemical (Ann Arbor, MI). Devazepide was purchased from Santa Cruz Biotechnology (Dallas, Texas). Fluorescein diacetate, propidium iodide, clofazimine, hexachlorophene, hydralazine hydrochloride, thioflavin S and Amicon Centrifugal Filter Unit were purchased from Sigma Aldrich (St. Louis, MO). Gibco Dulbecco’s Modified Eagle Medium (DMEM), HyClone™ Fetal Bovine Serum, Optical Adhesive Film and FITC-Annexin V were purchased from Thermo Fisher Scientific (Waltham, MA). All other chemicals for common buffers and solutions were from Sigma Aldrich.

### Plasmids and recombinant genes

Plasmid and expression procedures for the 1N4R tau and p-tau were previously described^59^. All constructs were verified by DNA sequencing. The final tau and p-tau expressed from the PIMAX system contained a 7-residue remnant from cloning: GSSPEQP at the N' end.

### Hyperphosphorylated tau expression, purification, and aggregation assays

Procedures for the preparation of PIMAX p-tau, as well as the conditions for p-tau aggregation assays can be found in reference^60^. The aggregation assays over the course of this project have been done with multi-plate readers from different sources. The absolute fluorescence units of thioflavin S recorded by each instrument might vary, but the trend of relative changes remained the same.

### High-throughput screening

A common mix of 6 µM p-tau, 20 mM Tris pH 7.4, 1 mM DTT, and 20 µM ThS was assembled and dispensed to 384-well low-volume plates (10 µl per well) using BioTek EL406 Washer, followed by the addition of 150 nl of the Prestwick Library compounds (final concentration of 30 µM per well) or DMSO in a Beckman Coulter Biomek FX^P^ workstation. Each plate was covered with an Optical Adhesive Film and incubated at 37 °C for about 10 min. ThS fluorescence was measured every 10 min at 440/490 nm for 16 h by Biotek Synergy Neo Plate Reader. Four plates were used to cover the 1280-compound library. The real-time kinetics of ThS signals were collected using Gen5 software. The net change of ThS of each reaction was calculated and was used to assess the standard deviation. The Z′-value of p-tau aggregation reactions was derived by first calculating the ThS net change over 16 h of no-protein, ThS-only reactions as the negative control (− c), and p-tau-containing reactions (with DMSO vehicle) as the positive control (+ c). The Z′-value was calculated by the equation:

$${\text{Z}}^{\prime } = {\text{ }}[1 - (3({\text{s}}_{{ + {\text{c}}}} + {\text{s}}_{{ - {\text{c}}}} )/\left| {\mu _{{ + {\text{c}}}} {-}{\text{ }}\mu _{{ - {\text{c}}}} } \right|)]$$

where σ_+c_, σ_-c_, µ_+c_, and µ_-c_ are the standard deviations (s) and the averages (µ) of the positive and negative controls^62^. All compounds showing greater than 3 standard deviations were picked for dose response curves (DRC). Compounds showing greater than 2 standard deviations and used for treating CNS conditions were selected for DRC as well. Those that showed significant deviation from no-compound controls were purchased from commercial venders and repeated for DRC.

### Cytotoxicity of p-tau

Cell viability assays were tested according to reference^60^ with modifications. PTAIs were typically added to cells along with (Fig. 4) or after p-tau was added to cells (Fig. 5). For PTAEs, 10 × concentration of p-tau and the test compounds were assembled for pre-aggregation (without heparin or ThS) for 48 h before adding 10 µl to 100 µl of cells in 96-well plates. Twenty-four or 48 h after the addition of protein/compound, cells were trypsinized and transferred to microcentrifuge tubes, and pelleted at 1000 × g for 5 min at room temperature for FDA, PI, annexin V, and ThS staining^60^. For PI/ThS double staining, cell pellets were resuspended in 20 mM Tris, pH7.4 buffer and incubated with 100 µM ThS for 5 min at room temperature. 5-µg/ml propidium iodide was then added to cell suspension for observation under a microscope.

## Supplementary information

Supplementary Table 1.

Supplementary Information.
