# Supplementary material for: Hyperphosphorylated tau aggregation and cytotoxicity modulators screen identified prescription drugs linked to Alzheimer's disease and cognitive functions
Source: Sci Rep. 2020 Oct 6;10:16551. doi: 10.1038/s41598-020-73680-2 (PMC7539012; doi:10.1038/s41598-020-73680-2)
Supplement: Supplementary file 2 — Supplementary Information. [file 41598_2020_73680_MOESM2_ESM.docx]

**Supplemental Information**

Hyperphosphorylated tau aggregation and cytotoxicity modulators screen identified prescription drugs linked to Alzheimer's disease and cognitive functions

**Mengyu Liu^1^, Thomas Dexheimer^2^, Dexin Sui^1^, Stacy Hovde^1^, Xiexiong Deng^1#^, Roland Kwok^3,4^, Daniel A Bochar^5^, and Min-Hao Kuo^1^***

From the ^1^Department of Biochemistry and Molecular Biology, ^2^Department of Pharmacology and Toxicology, Michigan State University, East Lansing, Michigan; ^3^Department of Biological Chemistry, and ^4^Department of Obstetrics and Gynecology, University of Michigan, Ann Arbor, Michigan; ^5^Cayman Chemical, Ann Arbor, Michigan

Running title: Alzheimer's disease drug and risk factor identification

^#^Present address: Molecular, Cellular, and Developmental Biology, University of Michigan, Ann Arbor, MI 48109-1085

*To whom correspondence should be addressed: Min-Hao Kuo, 603 Wilson Road, Room 401, Biochemistry Building, Department of Biochemistry and Molecular Biology, Michigan State University, East Lansing, MI 48824. E-mail: kuom@msu.edu. Telephone: 517-3550163

**Legends for supplemental table 1:**

**Supplemental Table 1.** Raw data of the 1280-compound screening for p-tau aggregation modulators. All compounds showing equal or greater than 2 SD from the mean change of ThS signals were repeated in the aggregation assays before dose response curves were assessed. Selective ones showing an effect close to 2 SD from the mean ThS net change, were reported to have relevance to Alzheimer's disease, and were brain permeant were also tested. R-(-)-apomorphine was within this group.

**Supplemental figures:**

**
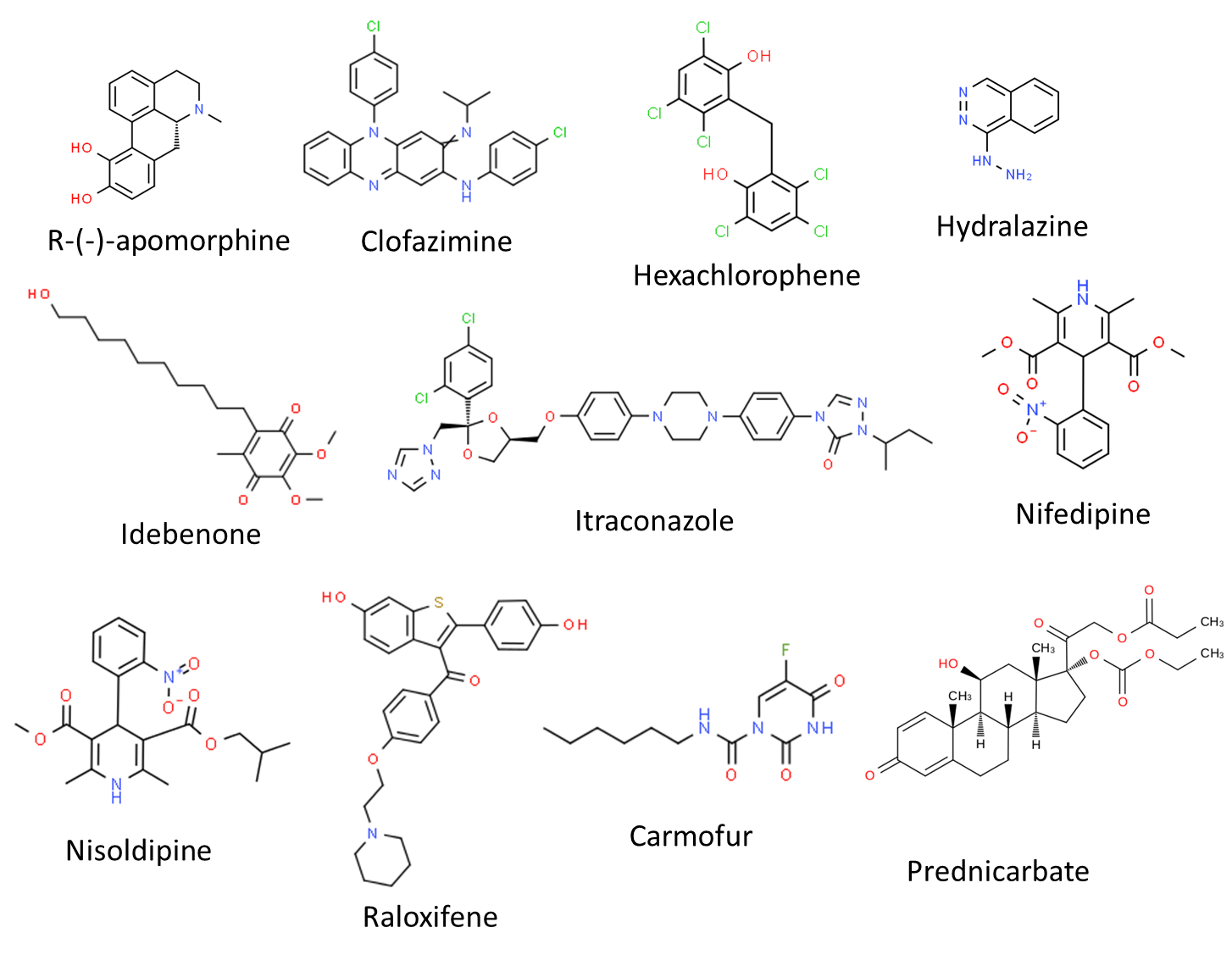
**

**Supplemental Figure 1.** Structures of PTAIs and PTAEs identified in the library screen.

**
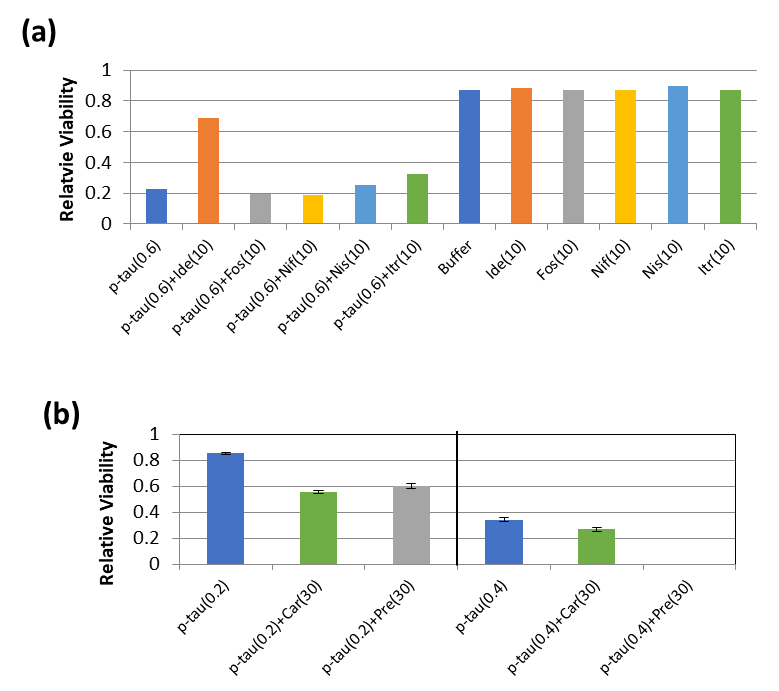
**

**Supplemental Figure 2.** Additional p-tau aggregation modulators control p-tau cytotoxicity. (a) Idebenone exhibits moderate cytoprotective activity against p-tau cytotoxicity. SH-SY5Y cells were treated with the indicated concentrations p-tau and compounds (in µM) for 20 hours before FDA/PI differential staining and microscopic quantification. (b) Prednicarbate and, to a lesser degree, carmofur exacerbated the p-tau cytotoxicity. 0.2 or 0.4 µM of p-tau and 30 µM of each compound were pre-incubated in aggregation reactions for 24 hours before the 20-hr cell treatment and cytotoxicity quantification. FDA and PI dual staining was performed to measure cellular viability. SH-SY5Y cells Compounds in panel (a) were added to cells along with p-tau without pre-incubation.

**
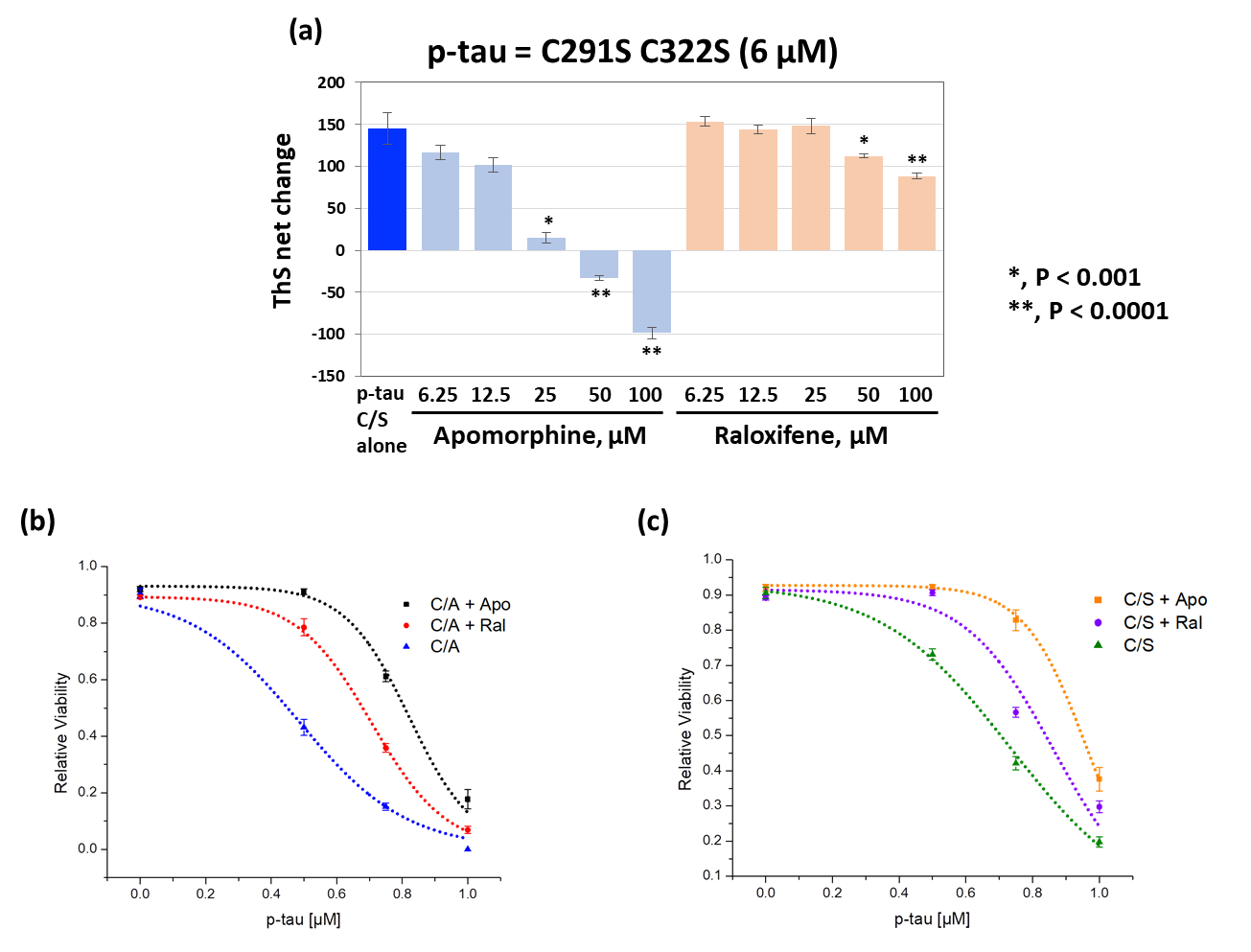
Supplemental Figure 3.** R-(-)-apomorphine and raloxifene control p-tau aggregation and cytotoxicity independently of the two cysteine residues of tau. (a) Net ThS fluorescence change of 12-hr p-tau (bearing C291A C322A mutations) aggregation reactions with p-tau alone (6 µM, dark blue column), or increasing concentrations of R-(-)-apomorphine (light blue columns) or raloxifene (pink columns). Error bars are standard deviations; n = 3. (b) Both C/A and C/S mutant p-tau are antagonized by R-(-)-apomorphine and raloxifene. SH-SY5Y cells were treated with 0, 0.5, 0.75, or 1 µM of either mutant p-tau. The cytoprotective activity of R-(-)-apomorphine (Apo) and raloxifene (Ral) was tested by including 10 µM of each compound in p-tau C/A or C/S treatment. Viability was quantified by fluorescence microscopy after FDA and PI differential staining. Error bars are standard deviation from one-tailed Student's t tests; n = 3.

**
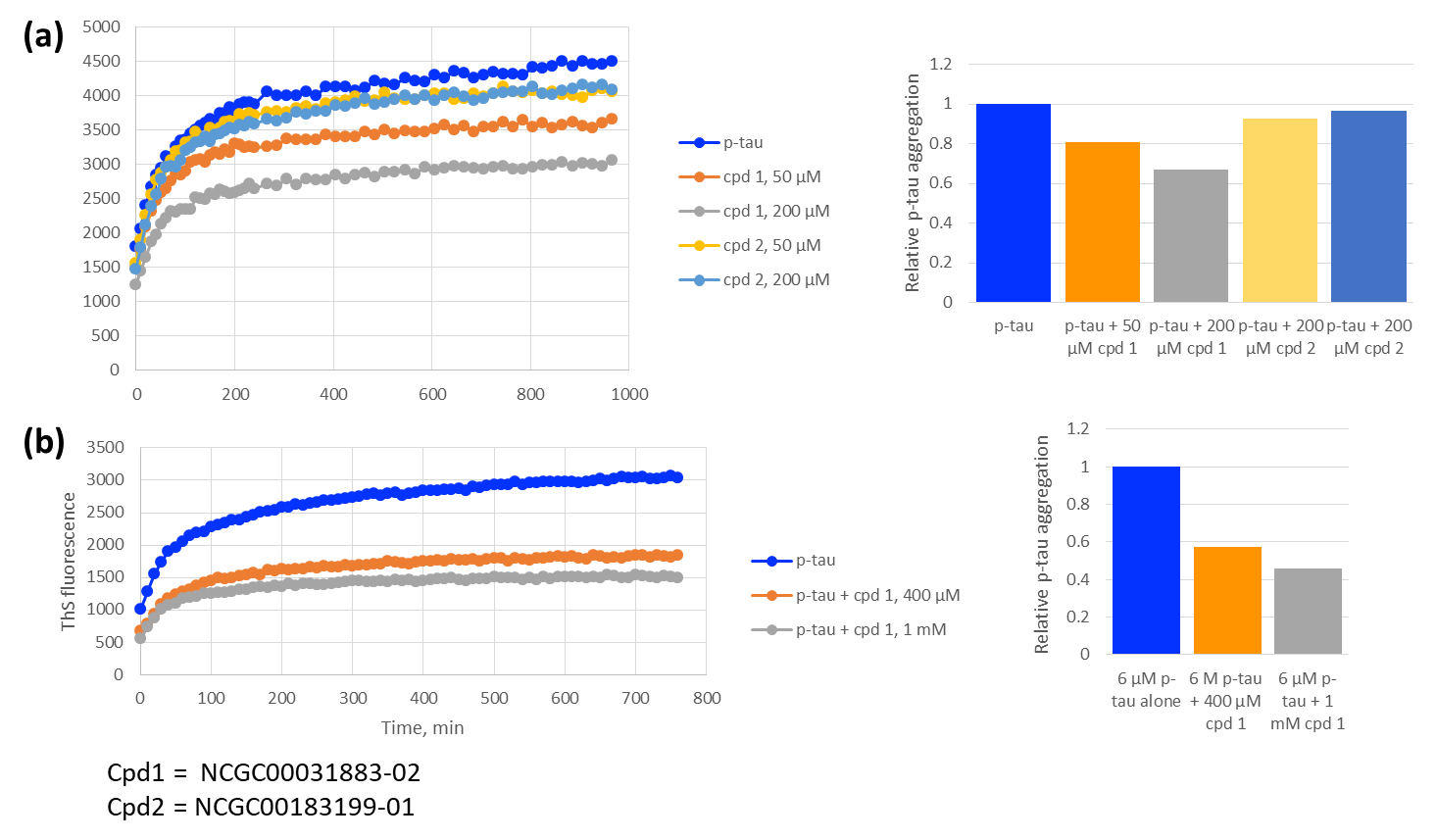
Supplemental Figure 4.** Tau aggregation inhibitors have a negligible or minor effect on p-tau aggregation. Two aminodienopyridazine compounds previously designated as potent tau aggregation inhibitors (84) were tested against p-tau aggregation, using the standard p-tau aggregation assay conditions established in this work. These two compounds were provided by Ajit Jadhav, National Center for Advancing Translational Sciences. Cpd1 = NCGC00031883-02; Cpd2 = NCGC00183199-01). The AC_50_ values for tau aggregation inhibition were reported to be 3.3 ± 0.2 µM and 6.8 ± 0.4 µM, respectively, when tested against 15 µM of K18 P301L aggregation induced by 40 µM of heparin (Probe Report ML103, National Center for Biotechnology Information, 2010). In each panel, the full-course kinetics of p-tau aggregation is shown on the left, and the net change of ThS fluorescence units is on the right. Note that these experiments were done with a different multi-plate reader, which consistently generated much higher fluorescence units than other models. Only the absolute fluorescence unit values differed; the trend and fold-of-change were essentially identical regardless of the fluorometers used.

**
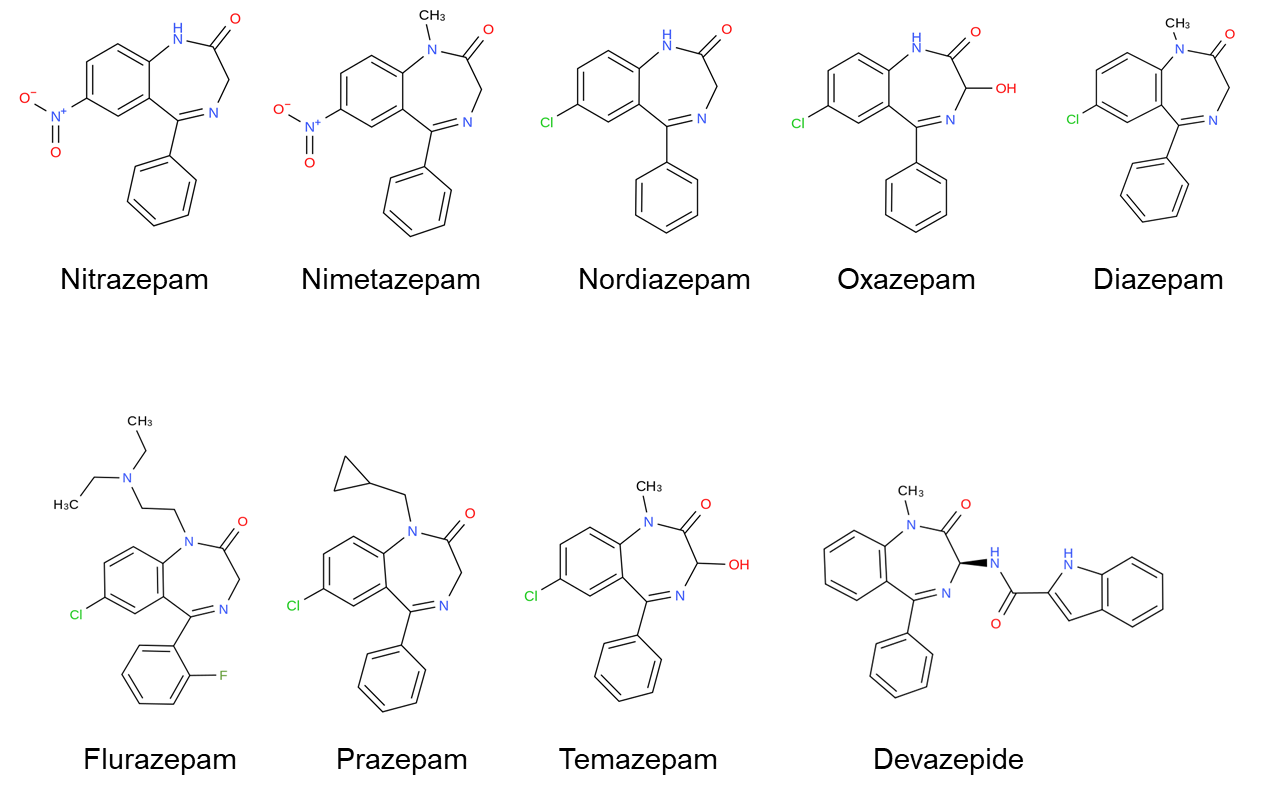
**

**Supplemental Figure 5.** Structures of benzodiazepines used in this study.
